# Hearing loss, sick leave, and disability pension: findings from the HUNT follow-up study

**DOI:** 10.1186/s12889-022-13760-2

**Published:** 2022-07-14

**Authors:** Astrid Ytrehus Jørgensen, Lisa Aarhus, Bo Engdahl, Bernt Bratsberg, Vegard Fykse Skirbekk, Ingrid Sivesind Mehlum

**Affiliations:** 1 Department of Occupational Medicine and Epidemiology, National Institute of Occupational Health, Oslo, Norway; 2 Department of Chronic Diseases and Ageing, Norwegian Institute of Public Health, Oslo, Norway; 3 The Ragnar Frisch Centre for Economic Research, Oslo, Norway; 4 Centre for Fertility and Health, Norwegian Institute of Public Health, Oslo, Norway

**Keywords:** Hearing loss, Sick leave, Disability pension

## Abstract

**Background:**

Evidence on the association between hearing loss and sick leave or disability pension is to a great extent based on few cross-sectional studies and remains unclear. We aim to assess the associations in a long-term follow-up population study.

**Methods:**

We used baseline data from a large population-based hearing study in Norway, the HUNT Hearing study (1996–1998). The sample included 21 754 adults (48.5% men, mean age at baseline 36.6 years). We used register data on sick leave and disability pension (1996–2011). Cox regression was used to assess the association between hearing loss at baseline (Pure tone average/PTA 0.5–4 kHz > 20 dB) and time to first physician-certified sick leave episode, as well as time to first disability pension payment.

**Results:**

Hearing loss at baseline (yes/no) was weakly associated with time to first physician-certified sick leave episode: Hazard ratio (HR) 1.2 (95% confidence interval (CI) 1.1–1.3). Restricting the exposed group to people with both hearing loss and tinnitus, the HR was slightly increased: 1.3 (95% CI 1.1–1.6). Hearing loss in 1996–1998 was also associated with time to first received disability pension: HR 1.5 (95% CI 1.3–1.8). Stronger associations were found for disabling hearing loss (PTA > 35). Restricting the exposure to hearing loss and tinnitus, the HR was increased: 2.0 (95% CI 1.4–2.8).

**Conclusions:**

This large population-based cohort study indicates that hearing loss is associated with increased risk of receiving disability pension, especially among younger adults and low educated workers. Hearing loss was weakly associated with sick leave.

## Summary

This paper evaluates the association between hearing loss and sick leave or disability pension. Our large population-based cohort study indicates that hearing loss is associated with increased risk of receiving disability pension and weakly associated with an increased risk of sick leave.

## Background

Hearing loss is regarded as one of the most prevalent disabilities [1] and a growing public health problem [2]. The WHO reports that throughout the world more than 430 million people experience disabling hearing loss, this number is estimated to reach 700 million by 2050 [3]. A recent large population study in Norway showed a prevalence of disabling hearing loss of 5.9 percent [4]. Hearing loss is also common within the working population. The prevalence of hearing loss among employed people in Norway has been showed to be 5.8%. For employed adults of 44 years or younger the prevalence was 1.3% and it was 11.3% for employed people above 44 years of age, showing that hearing loss is more common in older age groups. People with hearing loss have increased odds of non-employment and the association between hearing loss and non-employment has been showed to be stronger among younger adults compared to older adults [5].

Important risk factors for hearing loss include increasing age, genetic factors, ear disease and noise exposure [6]. Tinnitus is known as the conscious perception of an auditory sensation in the absence of an external stimulus. Examples of risk factors and associated characteristics include hearing loss, noise exposure, age, general health status, ear infections and head injury, along with certain diseases and medications [7].

A systematic review conducted in Sweden assessed 18 different studies concerning hearing difficulties, ear-related diagnoses and sickness absence or disability. The authors specify that their most striking finding was the low number of published studies about sick leave or disability pension due to hearing difficulties/diagnoses, considering the high prevalence of such hearing difficulties. They conclude that remarkably few studies were identified and that the results presented in them could not provide evidence for direction or magnitude of potential associations [8].

The present study aims to assess the association between hearing loss and sick leave or disability pension in a large long-term follow-up population study. We also aim to assess whether the associations are influenced by various factors, such as tinnitus, age, sex, or occupational class.

## Methods

### Participants

#### The HUNT study

The Trøndelag Health Study (The HUNT Study) is a longitudinal population health study that was conducted in the Norwegian county Trøndelag. Data from questionnaires, clinical measurements, and samples are included in the study, and lay a strong platform for population health studies. HUNT is regarded as one of the most comprehensive cohort studies that has been conducted, its data and samples were gathered from four separate waves (HUNT1, 2, 3, and 4) spanning the years 1984 to 2019.

#### The HUNT hearing studies

Audiometric investigations were performed in HUNT2 (1996–1998) and in HUNT4 (2017–2019). For the present study, the HUNT2 sample was used for follow-up analyses. Participants in the HUNT2 Hearing study came from 17 of the county’s 24 municipalities. The participation rate was 63%, and altogether 50 560 persons attended [4]. HUNT2 Hearing will be referred to as “HUNT” hereafter for the sake of simplicity.

#### Present study sample (HUNT)

From the total HUNT population of 50 560 persons, we excluded persons in the following order: persons not in the age-range 20–49 years (*N* = 25 118), non-employed persons at baseline (*N* = 3293), persons with missing audiometric data (*N* = 98) and persons with missing questionnaires (*N* = 297). Non-employed persons at baseline were excluded to ensure that all participants were in work and at risk for sick leave or disability pension at the starting point of the follow-up period. The final sample included 21 754 subjects.

## Measurements

### Exposure variables

#### Hearing loss

The HUNT hearing study included pure-tone audiometry, otoscopy, and a comprehensive questionnaire. Pure-tone air-conduction hearing thresholds levels (HTLs) were measured in accordance with ISO 8253–1 [9], with fixed frequencies at the eight test frequencies 0.25, 0.5, 1, 2, 3, 4, 6 and 8 kHz, using an automatic procedure (“push the button when you hear a sound”). Masking was not applied, and bone conduction thresholds were not assessed. The elderly or those who were unable to follow the automatic procedure were provided manual audiometry. The audiometry procedure has been described in detail previously [4]. We used the average of the hearing thresholds measured at frequencies 0.5, 1, 2, and 4 kHz in the best hearing ear with the Global Burden of Disease (GBD) definition of hearing loss to construct a categorical variable with normal hearing (hearing threshold < 20 dB) as the reference category [0], mild hearing loss (20–34 dB) [1] or disabling hearing loss (≥ 35 dB) [2]. We also constructed a dichotomous variable with normal hearing as the reference category and any hearing loss (> 20 dB) as exposed.

#### Tinnitus

We constructed a dichotomous variable to compare participants with hearing loss and tinnitus (exposed) to normal hearing and no tinnitus as reference category. For the construction of this variable, we used the following question from the HUNT2 questionnaire: «Have you experienced ringing in your ears/tinnitus during the last 12 months?». Participants with missing values were excluded from this particular analysis.

### Outcome variables

#### Sick leave and disability pension

We obtained yearly data on physician-certified sick leave (episodes of more than 16 days) and disability pension from 1996 to 2016 from Statistics Norway. Based on the personal identification number given to all Norwegian citizens, data from Statistics Norway were linked on an individual level with the data from the HUNT survey. Identification numbers were removed before the researchers were given access to the matched data material. We linked the HUNT data with individual records covering sick leave episodes between 1998 and 2016.

### Potential effect modifiers or confounders

#### Age and sex

In all analyses, we adjusted for age and gender.

#### Education

Data from Statistics Norway on education was used to construct this variable. Educational level was divided into 4 groups: primary education, secondary education, university < 4 years, university ≥ 4 years.

A dichotomous variable was created by combining primary school and secondary school to give the group “lower education” and university < 4 years with university ≥ 4 years to give the group “higher education” [5]. This dichotomous variable was used in the analyses. We excluded persons with missing data (*N* = 13). We Adjusted for education in all analyses.

#### White-collar/blue-collar

Occupational codes from Statistics Norway were available from 1990 and 1980. We used NYK codes (Nordisk yrkesklassifisering; “Nordic Occupational Classification), based on the International Standard Classification of Occupations, ISCO-58 [10]. At the one-digit level, the occupations are divided into 12 major groups: 0 = Technical, physical science, humanistic and artistic work; 1 = Administrative, executive and managerial work; 2 = Clerical work; 3 = Sales work; 4 = Agriculture, forestry and fishermen’s work; 5 = Mining and quarrying work etc.; 6 = Transport and communication work; 7 and 8 = Manufacturing and construction work; 9 = Service work; A = Military work; X = Occupation not reported. We categorised the occupational codes 0–3 as “mainly white-collar workers”, and the codes 4–9 and A as “mainly blue-collar workers”. Persons who were not registered with an occupational code (not working or lack of registrations/missing data) and persons with occupational code X (occupation not reported), were excluded from this specific analysis (*N* = 6495).

### Statistical analyses

We used STATA version 17.0. Statistical tests were calculated at a 95% confidence interval.

#### Hearing loss and sick leave

We assessed the association between hearing loss in 1996–1998 and time to first physician-certified sick leave episode (1996–2011) by using Cox Proportional Hazards Regression. We chose to end the follow-up period at the year 2011 because a Norwegian pension reform was introduced that year. It allowed people over 62 years of age to combine work and retirement, which would have made the time period before and after 2011 non-comparable in terms of the censoring at time of retirement. We analysed time in calendar days to incident sick leave during the follow-up period, starting at the date of the hearing test in 1996–1998 (baseline), ending at the end of 2011.

We used data from Statistics Norway (SSB) on employment status to construct categories for censoring. The SSB data on employment status were categorized in 5 groups: wage earner, self-employed, unemployed, outside of workforce (retired, disabled, student, homemaker) or in labour market programs. Labour market programs are part of the Norwegian social welfare system aiming to improve chances of finding employment, offering job-finding measures, work experience and job training.

Participants were censored at year of leaving employment (unemployment, outside workforce, or in labour market programs), at date of death or at the end of the follow-up period with a mean follow-up time of 6.7 years and a maximum of 15.0 years. This summed up to a total follow-up time of 145 723 person-years.

#### Hearing loss and disability pension

We assessed the association between hearing loss in 1996–1998 and time to first pay out of disability pension (1996–2011) using the same method as for sick leave. Participants were censored at year of retirement from work, at year of death or at the end of the follow-up period with a mean follow-up time of 14.1 years and a maximum of 15 years and a total follow-up time of 304 000 person-years.

Finally, we performed subgroup analyses investigating the risk of physician-certified sick leave and the risk of receiving disability pension when the exposed group was defined as persons with both hearing loss and tinnitus.

For the above analyses, we adjusted for age (using a continuous age variable), sex, and education. We chose not to adjust for cardiovascular risk factors or smoking, because although many cardiovascular risk factors are associated with hearing loss, the effects have been shown to be small [11]. We also assessed whether the associations between hearing loss and sick leave or disability pension were modified by age, sex, education, or occupational class by performing stratified analyses and by testing interaction terms with the variable for hearing loss as a continuous variable (hearing loss *age, hearing loss *sex, hearing loss *education, hearing loss *occupational class). We have only performed interaction analysis between hearing loss and the mentioned variables, not for the group with hearing loss and tinnitus, due to the way this variable was constructed. The post-estimation proportional hazards test was used to test for proportional hazards. The proportional hazards assumption was met in all models.

## Results

### Descriptive results

#### Characteristics of the sample

The final sample included 21 754 persons (48.5% men, mean age at entry 36.6 years). The participants with any hearing loss were older than those with normal hearing (mean age 41.6 years vs. 36.5 years), and the proportion of males was higher (62.9% vs. 48.2%) (Table 1). The groups were similar with regards to the proportion of participants with higher education (64.4% vs. 60.7%). Tinnitus was more frequent among participants with hearing loss than those with normal hearing (36.5% vs. 9.3%). During follow-up, 15 984 was registered with at least one sick leave episode and 2091 with a pay out of disability pension.

**Table 1:** Background data of the total sample (age range 20–49 years), the HUNT study (1996–1998), Norway

	**Total**	**Normal**	**Any**	**Mild**	**Disabling**	**Any hearing loss**
	**sample**	**hearing**	**hearing loss**	**hearing loss**	**hearing loss**	** + tinnitus**
	**(*N* = 21 754)**	**(*N* = 21 231)**	**(*N* = 523)**	**(*N* = 433)**	**(*N* = 90)**	**(*N* = 182)**
Mean age at baseline—mean, (SD)	36.6 (8.2)	36.5 (8.2)	41.6 (7.0)	41.9 (6.9)	40.1 (7.3)	41.0 (7.2)
Men N, (%)	10 554 (48.5)	10 225 (48.2)	329 (62.9)	278 (64.2)	51 (56.7)	127 (69.8)
Women N, (%)	11 200 (51.5)	11 006 (51.8)	194 (37.1)	155 (35.8)	39 (43.3)	55 (30.2)
Younger adults (< 35 yr at baseline) N, (%)	9402 (43.2)	9391 (43.8)	101 (19.3)	77 (17.8)	24 (26.7)	38 (20.9)
Older adults (> 35 year at baseline) N, (%)	12 352 (56.8)	11 930 (56.2)	422 (80.7)	356 (82.2)	66 (73.3)	144 (79.1)
High education N, (%)	13 222 (60.8)	12 885 (60.7)	337 (64.4)	271 (62.6)	66 (73.3)	122 (67.0)
Low education N, (%)	8532 (39.2)	8346 (39.3)	186 (35.6)	162 (37.4)	24 (26.7)	60 (33.0)
White-collar N, (%)	6374 (41.8)	6241 (42.1)	133 (31.3)	113 (31.3)	20 (31.3)	42 (27.6)
Blue-collar N, (%)	8885 (58.2)	8593 (57.9)	292 (68.7)	248 (68.7)	44 (68.7)	110 (72.4)
Mean hearing threshold (dBHL)	3.6	2.9	29.8	25.3	51.3	30.6
Sample proportion with tinnitus (% of 20 784)	9.9	9.3	36.5	36.2	37.8	100
Participants with at least 1 episode of sick leave N, (%)	15 984 (73.5)	15 580 (73.4)	404 (77.2)	338 (78.1)	66 (73.3)	143 (78.6)
Participants that received disability pension N, (%)	2091 (9.6)	1997 (9.4)	94 (18.0)	74 (17.1)	20 (22.2)	38 (20.9)

*N* = 15,259 for white-/blue-collar

*N* = 20,784 for tinnitus variable

Most women (in all the different strata) had at least one episode of sick leave during the follow-up period (81–89%), whereas for men the proportion varied between 63–74% (Table 2). The group with the highest level of sick leave, was women with any hearing loss and tinnitus. The differences in absolute numbers for sick leave episodes were smaller between other dichotomies (younger/older adults, high/low education, white-/blue-collar), than between men and women.

**Table 2:** Participants with at least 1 episode of sick leave during follow-up, the HUNT study (1996–1998), Norway

	**Total**	**Normal**	**Any**	**Mild**	**Disabling**	**Any hearing loss**
	**sample**	**hearing**	**hearing loss**	**hearing loss**	**hearing loss**	** + tinnitus**
	**(*N* = 21 754)**	**(*N* = 21 231)**	**(*N* = 523)**	**(*N* = 433)**	**(*N* = 90)**	**(*N* = 182)**
	**N (%)**	**N (%)**	**N (%)**	**N (%)**	**N (%)**	**N (%)**
**Total sample**	15 984 (73.5)	15 580 (73.4)	404 (77.2)	338 (78.1)	66 (73.3)	143 (78.6)
**Stratified analysis**						
Men	6948 (65.8)	6712 (65.6)	236 (71.7)	204 (73.4)	32 (62.7)	94 (74.0)
Women	9036 (80.7)	8868 (80.6)	168 (86.6)	134 (86.5)	34 (87.2)	49 (89.1)
Younger adults < 35 yr at baseline	6753 (71.8)	6679 (71.1)	74 (73.3)	57 (74.0)	17 (70.8)	29 (76.3)
Older adults > 35 year at baseline	9231 (74.7)	8901 (74.6)	330 (78.2)	281 (78.9)	49 (74.2)	114 (79.2)
High education	9669 (73.1)	9408 (73.0)	261 (77.4)	214 (79.0)	47 (71.2)	98 (80.3)
Low education	6315 (74.0)	6172 (74.0)	143 (76.9)	124 (76.5)	19 (79.2)	45 (75.0)
White-collar	4685 (73.5)	4586 (73.5)	99 (74.4)	84 (74.3)	15 (75.0)	31 (73.8)
Blue-collar	6629 (74.6)	6396 (74.4)	233 (79.8)	201 (81.0)	32 (72.7)	91 (82.7)

*N* = 15,259 for white-/blue-collar

*N* = 20,784 for tinnitus variable

The group with the lowest proportion of people receiving disability pension during follow up was young adults with normal hearing (2.7%), followed by young adults with mild hearing loss (6.5%) and men with normal hearing (6.9%) (Table 3). The groups that showed the highest proportion of receiving disability pension were people with low education and disabling hearing loss (37.5%), women with disabling hearing loss (30,8%) and women with any hearing loss and tinnitus (30.9%). These groups are, however, quite small. The largest differences between strata in absolute numbers for how many people received disability pension during the follow up period, can be seen between young and old adults, and between high and low education for people with hearing loss.

**Table 3:** Participants that received disability pension during follow-up, the HUNT study (1996–1998), Norway

	**Total**	**Normal**	**Any**	**Mild**	**Disabling**	**Any hearing loss**
	**sample**	**hearing**	**hearing loss**	**hearing loss**	**hearing loss**	** + tinnitus**
	**(*N* = 21 754)**	**(*N* = 21 231)**	**(N = 523)**	**(*N* = 433)**	**(*N* = 90)**	**(*N* = 182)**
	**N (%)**	**N (%)**	**N (%)**	**N (%)**	**N (%)**	**N (%)**
**Total sample**	2091 (9.6)	1997 (9.4)	94 (18.0)	74 (17.1)	20 (22.2)	38 (20.9)
**Stratified analysis**						
Men	756 (7.2)	709 (6.9)	47 (14.3)	39 (14.0)	8 (15.7)	21 (16.5)
Women	1335 (11.9)	1288 (11.7)	47 (24.2)	35 (22.6)	12 (30.8)	17 (30.9)
Younger adults < 35 yr at baseline	259 (2.8)	250 (2.7)	9 (8.9)	5 (6.5)	4 (16.7)	5 (13.2)
Older adults > 35 year at baseline	1832 (14.8)	1747 (14.6)	85 (20.1)	69 (19.4)	16 (24.2)	33 (22.9)
High education	1224 (9.3)	1177 (9.1)	47 (13.9)	36 (13.3)	11 (16.7)	22 (18.0)
Low education	867 (10.2)	820 (9.8)	47 (25.3)	38 (23.5)	9 (37.5)	16 (26.7)
White-collar	685 (10.7)	663 (10.6)	22 (16.5)	18 (15.9)	4 (20.0)	8 (19.0)
Blue-collar	1014 (11.4)	959 (11.2)	55 (18.8)	47 (19.0)	8 (18.2)	26 (23.6)

*N* = 15,259 for white-/blue-collar

*N* = 20,784 for tinnitus variable

### Results from Cox regression analyses

#### Hearing loss and sick leave

There was a weak association between any hearing loss in 1996–1998 and time to first sick leave episode for the total sample (Table 4). Stratified analyses showed weak associations between any hearing loss and sick leave for women, younger adults, older adults, and people with both low and high education. The same was true for associations with mild hearing loss for women, both younger and older adults, higher educated and people with blue-collar occupations. People who had both hearing loss and tinnitus had a slightly higher risk of sick leave (Table 4). As to the tests for effect modification, there were two statistically significant interaction terms: hearing loss (continuously scored) *sex (p = 0.003) and hearing loss (continuously scored)*white/blue-collar occupation (p = 0.007). In other words, the association between hearing loss and sick leave was modified by sex (stronger association among women than men) and by occupational field (stronger association among white-collar workers than blue-collar workers).

**Table 4:** Associations between hearing loss at baseline and sick leave during follow-up among employed persons, The HUNT Study, Norway

	**Any hearing loss**	**Mild hearing loss**	**Disabling hearing loss**	**Any hearing loss + tinnitus**
	**(*N* = 523)**	**(*N* = 433)**	**(*N* = 90)**	**(*N* = 182)**
	**Hazard ratio (95% CI)**	**Hazard ratio (95% CI)**	**Hazard ratio (95% CI)**	**Hazard ratio (95% CI)**
**Total sample (*N* = 21 754)**	1.2 (1.1–1.3) *	1.2 (1.1–1-3) *	1.1 (0.8–1.4)	1.3 (1.1–1.6) *
**Stratified analysis**				
Men	1.1 (1.0–1.3)	1.1 (1.0–1.3)	1.0 (0.7–1.4)	1.2 (1.0–1.5) *
Women	1.2 (1.1–1.4) *	1.3 (1.1–1.5) *	1.2 (0.8–1.6)	1.5 (1.1–2.0) *
Younger adults (< 35 yr at baseline)	1.3 (1.0–1.6) *	1.3 (1.0–1.7) *	1.2 (0.8–2.0)	1.5 (1.0–2.2) *
Older adults (> 35 year at baseline)	1.1 (1.0–1.3) *	1.2 (1.0–1.3) *	1.0 (0.8–1.3)	1.3 (1.1–1.5) *
High education	1.2 (1.0–1.3) *	1.2 (1.1–1.4) *	1.0 (0.7–1.3)	1.3 (1.1–1.6) *
Low education	1.2 (1.0–1.4) *	1.1 (1.0–1.4)	1.5 (1.0–2.4)	1.3 (1.0–1.8) *
White-collar	1.1 (0.9—1.4)	1.2 (0.9—1.5)	1.0 (0.6—1.7)	1.2 (0.9–1.8)
Blue-collar	1.1 (1.0—1.3)	1.1 (1.0—1.3) *	1.0 (0.7–1.4)	1.3 (1.1–1.6) *

All analyses are adjusted for age, sex, and education

In the sex stratified analyses, the estimates are adjusted for age and education

In the age stratified analyses, the estimates are adjusted for sex and education

In the education stratified analyses, the estimates are adjusted for sex and age

*CI*   confidence interval

^*^ = *p* ≤ 0.05

#### Hearing loss and disability pension

Hearing loss in 1996–1998 was associated with getting a disability pension during the follow-up period (Table 5), statistically significant for all groups in the stratified analyses, except blue-collar occupations. Stratified analyses showed associations between any hearing loss and time to receiving disability pension for all groups, except blue-collar occupations. Young adults with any hearing loss had twice the risk of disability uptake compared to those with normal hearing. Participants with disabling hearing loss had two times the risk of receiving disability pension compared with normal hearing participants, or more. This was true for all the groups in the stratified analyses, except for people in blue-collar occupations. However, there is some degree of uncertainty, as the confidence intervals are somewhat wide. Younger adults and low educated participants with disabling hearing loss had a more than threefold increased risk of receiving disability pension. The stratified analyses showed a stronger association between hearing loss and receiving disability pension for women compared to men in every stratum of hearing loss.

**Table 5:** Associations between hearing loss at baseline and disability pension during follow-up among employed persons, The HUNT Study, Norway

	**Any hearing loss**	**Mild hearing loss**	**Disabling hearing loss**	**Any hearing loss + tinnitus**
	**(*N* = 523)**	**(*N* = 433)**	**(*N* = 90)**	**(*N* = 182)**
	**Hazard ratio (95% CI)**	**Hazard ratio (95% CI)**	**Hazard ratio (95% CI)**	**Hazard ratio (95% CI)**
**Total sample (*N* = 21 754)**	1.5 (1.3–1.8) *	1.4 (1.1–1-7) *	2.3 (1.6–3.2) *	2.0 (1.4–2.8) *
**Stratified analysis**				
Men	1.4 (1.1–1.8) *	1.3 (1.0–1.7)	2.1 (1.3–3.6) *	1.7 (1.1–2.7) *
Women	1.6 (1.3–2.0) *	1.5 (1.1–1.9) *	2.4 (1.5–3.8) *	2.4 (1.5–3.9) *
Younger adults < 35 yr at baseline	2.2 (1.4–3.7) *	1.8 (1.0–3.4)	3.7 (1.7–8.4) *	5.3 (2.2–12.8) *
Older adults > 35 year at baseline	1.4 (1.2–1.7) *	1.3 (1.1–1.6) *	2.1 (1.4–3.0) *	1.8 (1.3–2.6) *
High education	1.4 (1.1–1.7) *	1.2 (0.9–1.6)	2.0 (1.3–3.0) *	1.8 (1.2–2.8) *
Low education	1.7 (1.4–2.3) *	1.6 (1.2–2.1) *	3.3 (1.8–6.2) *	2.2 (1.3–3.6) *
White-collar	1.6 (1.2–2.3) *	1.6 (1.1–2.2) *	2.1 (0.9–4.7)	1.7 (0.9–3.5)
Blue-collar	1.2 (1.0–1.5)	1.1 (0.9–1.4)	1.8 (1.1–2.8) *	1.9 (1.3–2.8) *

All analyses are adjusted for age, sex, and education

In the sex stratified analyses, the estimates are adjusted for age and education

In the age stratified analyses, the estimates are adjusted for sex and education

In the education stratified analyses, the estimates are adjusted for sex and age

*CI*   confidence interval

^*^ = *p* ≤ 0.05

There were two statistically significant interaction terms: hearing loss (continuously scored)*age (*p* = 0.003) and hearing loss (continuously scored)*education (*p* = 0.001).In other words, the association between hearing loss and disability pension was modified by age group (stronger association among younger than older adults) and by education (stronger association among lower educated than higher educated persons).

Among people with both hearing loss and tinnitus, the odds of receiving disability pension during the follow-up period were doubled. For younger adults the risk was more than five times higher than for people with normal hearing. (Table 5).

## Discussion

### Main findings

This study showed an association between hearing loss and time to first physician-certified sick leave episode, and with time to receiving disability pension. Hearing loss in 1996–1998 was weakly associated with time to first physician-certified sick leave episode. Restricting the exposed group to people with both hearing loss and tinnitus, the HR was slightly increased. Hearing loss in 1996–1998 was also associated with time to first received disability pension pay. People with disabling hearing loss had more than a twofold increase in odds of receiving disability pension compared with people with no hearing loss. Younger adults with hearing loss and tinnitus had a more than five times increased risk of receiving disability pension.

### Evaluation of results and comparisons with other studies

#### Hearing loss and sick leave

Our study showed that there was a statistically significant but weak association between hearing loss (yes/no) and time to first physician-certified sick leave episode. A systematic review concluded that remarkably few studies on this topic were identified and pointed out a lack of follow-up studies [8]. To our knowledge, large population-based studies with a follow-up design examining this association have not been undertaken previously. However, some smaller studies have reported positive associations. For example, a questionnaire-based study of 210 participants showed that sick leave due to distress occurred more often in the hearing impaired than in employees with normal hearing [12]. Another survey-based cross-sectional study, of the causes and severity of injuries in 880 construction workers, showed an association between hearing disorders and long-term sick leave [13]. A study looking at speech-in-noise measurements and self-reports of sick leave in 748 workers, showed that decreasing hearing ability in noise significantly increased the odds for sick leave of more than 5 days [14].

#### Hearing loss, tinnitus and sick leave

Our study showed that subgroup analysis including people who had both hearing loss and tinnitus, showed a somewhat increased risk of sick leave. A paper by Holgers et al. aimed to investigate risk factors for incapacitating tinnitus by measuring absence from work relating to tinnitus [15]. The study sample was relatively small. They showed that 18 of the 79 included patients had been absent from work due to tinnitus during an 18-month period and concluded that the main predictors of tinnitus leading to sickness absence were depression and physical immobility and that these factors were stronger than hearing loss. Friberg et al. showed that sick leave spells due to tinnitus diagnoses tended to be long, often lasting an entire year, in a study of sick leave due to otoaudiological diagnoses [16].

#### Hearing loss and work disability pension

We found a higher risk for receiving disability pension in the group with hearing loss, particularly for persons with disabling hearing loss. Helvik et al. investigated hearing loss and the risk of disability pension in Norway in 2013 [17]. Helvik et al. also used a study sample from HUNT2, however the current study has a different design and the cases (participants receiving disability pension) included in the Helvik-study are not included in the current study. They showed that hearing loss diagnoses are rarely reported as the main cause in disability diagnostics, but that the degree of hearing loss increased the risk of being granted with disability pension with diagnoses not related to hearing loss. A more recent, survey-based study of 2407 Danish respondents showed a higher likelihood of receiving disability pension among people with hearing impairment [18]. The negative aspects of working life experienced by people with hearing loss, are likely to influence receipt of disability pension. Communication is an important aspect of work tasks in occupations that involve contact with customers and clients, which would likely affect people with hearing loss. Another aspect of modern working life that has been shown to have a negative impact on employees with hearing loss is open plan workspaces [19]. We show that women with hearing loss have a higher risk of receiving disability pension compared to men. Occupations in which communication is an important factor of work, such as health and social work, often have a higher proportion of female compared to male workers [5]. This may be part of the reason behind why there were some indications that women are more vulnerable to disability pension than men with hearing loss.

#### Work disability pension versus sick leave

From our findings, we saw that there was a stronger association between hearing loss and receiving disability pension than there was between hearing loss and physician-certified sick leave during the follow-up period. Speculating on the causes of this disparity, it could be due to the relative commonness of sick leave compared to disability pension. Having an episode of sickness absence from work is relatively common, whereas receiving disability pension less so. Most people on sick leave do not receive a disability pension later in life. Gustafsson et al. assessed the risk of future disability pension among people with sick leave due to otoaudiologcal diagnoses (OAD) compared to other sickness absentees and found more than 40% higher risk among those on sick leave due to OAD [20]. Although their study included participants with hearing loss and tinnitus, it is not directly comparable to our study as it also included vestibular diagnoses.

#### Vulnerable groups

We found that younger adults and people with low education were at increased risk of disability pension. The finding of a greater effect of hearing loss among young adults corresponds with the findings of another study evaluating hearing loss and mental health [21], which showed that effects of hearing loss were stronger among young people. Further research that investigates groups that are more vulnerable for receiving disability pension among working hearing-impaired people would be of interest to target future interventions in the workplace effectively.

#### Interpretations of the findings

We can only speculate about the underlying cause of our findings. Impaired hearing affects communication and psychosocial functioning [6]. A recent study of hearing loss and work participation factors showed a negative association between hearing loss and workability, as well as work role functioning [22]. Poorer hearing ability in noise is associated with increased need for recovery after work [23], while increased need for recovery is again associated with sick leave [24].

#### Implications of the findings

Our findings indicate that effort should be made to implement preventive measures for hearing impaired in the workplace. A greater emphasis has been placed on adapting workplaces to accommodate for hearing impairment. Moreover, there has been an increase in the education level of people with hearing impairment in recent times [25]. Additionally, there has been an increased digitalisation of work and a significant change from cellular to open-plan workspaces [26]. Increased digitalisation reduces reliance on oral communication, which is a positive development for hearing impaired workers. Open-plan offices, on the other hand, have been shown to have a negative impact on employees with hearing loss [19].

### Strengths and limitations of the study

#### Strengths

Strengths of the present study includes the large number of participants, the long follow-up period, register-based dates of sick leave and disability, standardized audiometric measurements, and the representative population sample from Trøndelag County, which has been shown to be representative of Norway [27].

#### Weaknesses

It is well known that some subgroups, such as low socioeconomic groups and those in poor physical condition, have lower participation rates in health surveys [28]. This may have underestimated our results. Only persons in employment were included at baseline and it may be that people with hearing loss that are in work are healthier or have other positive characteristics compared with those that are unemployed. This potential selection bias may give an underestimation of our results. We were unable to focus our research on the precise causes of sick leave, as information about the diagnoses behind the episodes were not available. It would have been interesting to evaluate if there was a stronger association between hearing loss and sick leave for certain groups of diagnoses, for instance diagnoses related to depression or burnout. Information on occupational class was registered in the 1990s, for persons with missing registrations in the -90 s data was imputed from the1980s. However, most people are expected to continue working in either blue-collar or white-collar occupations.

## Conclusion

This large population-based cohort study indicates that hearing loss is weakly associated with later physician-certified sick leave and with receiving disability pension. These associations were stronger for participants with both hearing loss and tinnitus. For disability pension, women, younger adults, and low educated workers seem to be more vulnerable.

## Data Availability

The data that support the findings of this study are available from Statistics Norway and the HUNT Study and were collected in accordance with national guidelines. Restrictions apply to the availability of these data, which were used under license for the current study, and so are not publicly available. Contact the corresponding author for more details on data availability.

## Abbreviations

CI: Confidence interval
dB: Decibel
GBD: Global burden of disease
HR: Hazard ratio
HUNT: The Trøndelag Health Study
NYK: Nordic Occupational Classification
PTA: Pure tone average
SSB: Statistics Norway
WHO: World Health Organization
